# Patient perceptions of the readability and helpfulness of bilingual clinical forms: a survey study

**DOI:** 10.1186/s12909-023-04519-3

**Published:** 2023-08-25

**Authors:** Romana Muller, Lynda Tierney Konecny

**Affiliations:** 1 Missouri School of Dentistry & Oral Health, St. Louis Dental Center, A.T. Still University1500 Park Ave, St. Louis, MO 63104 USA; 2 College of Graduate Health Studies, A.T. Still University, 800 W Jefferson St, Kirksville, MO 63501 USA

**Keywords:** Dental school, Language barriers, Limited English proficiency, Refugees and immigrants, Translation of dental forms

## Abstract

**Background:**

Patients with limited English proficiency (LEP) are rarely provided with translated clinical materials. Typically, healthcare clinics cite high costs of translation and lack of professional translators as barriers to this service. The purpose of the current study was to investigate the perceptions of LEP dental patients regarding the readability, understanding, and helpfulness of translated clinical forms produced by dental student doctor translators.

**Methods:**

We used a survey design and convenience sampling to recruit LEP patients from a dental school clinic. Participants completed a 9-question (8 Likert-type items and 1 open-ended item) paper survey about translated forms. The bilingual survey was a combination of English and 8 other languages (Arabic, Dari, Pashto, Russian, Spanish, Ukrainian, Urdu, or Vietnamese) and assessed the type of form received; self-reported literacy; design, readability, and helpfulness of the form; overall understanding of the form; understanding of medical and dental terms; helpfulness for patient-provider communication; and comfort level with dental care after receiving the form. Demographic characteristics of participants were collected from the clinic’s electronic health record. Survey responses were analyzed descriptively, and Spearman’s correlation was used to examine the relationship between outcomes.

**Results:**

Ninety-seven LEP patients (61.9% [60] female, 78.4% [70] Spanish speakers) completed 140 surveys for various translated forms in Dari, Pashto, Spanish, Urdu, or Vietnamese. Participants positively rated translated clinical forms: range, 50.4% (70) for design of the form to 80.0% (112) for comfort level with dental care after receiving the form. For the open-ended item, participants indicated the translations were good, and no improvements were needed. They also thought providing the form was evidence of good customer service. When examining relationships between outcomes, positive correlations were found between self-reported literacy and readability (Spearman *r* = .57, *P* < .001), overall understanding and understanding of medical and dental terms (Spearman *r* = .58, *P* < .001), and type of form and helpfulness for patient-provider communication (Spearman *r* = .26, *P* = .005).

**Conclusions:**

Study results suggested the translated clinical forms were perceived as helpful and beneficial by LEP dental patients. Similar approaches should be considered to reduce language barriers in healthcare.

## Background

According to recent census data, 21% (60.6 million) of the US population speaks a language other than English, and 22% of those do not speak English well or at all [1]. Further, as many as 350 languages are spoken among ethnically diverse individuals residing in the United States [2]. Therefore, limited English proficiency (LEP) patients may have language barriers that contribute to medical errors and negatively affect patient-provider communication, healthcare access, and health outcomes [3, 4]. Although federal mandates require healthcare clinics receiving federal funds to provide free language assistance to LEP patients [5], assistance is usually limited because of financial and organizational barriers [6], and translated materials and clinical forms are often lacking [3, 4, 7–9].

Few studies have investigated the availability of translated materials [7, 8] even though translations of procedural processes and information for caregivers have been reported as some of the most needed materials [10]. In one study, providers used Google Translate for healthcare materials, but only 57.7% of 260 medical phrases were accurate [11]. In the dental setting, the language preference of LEP patients is not typically recorded [9], and providers tend to be unaware of those preferences because the patients are unable to communicate them [10]. Further, because of difficulties communicating with LEP patients, dental specialists often chose a simpler treatment option, such as tooth removal, instead of a more complex treatment that requires multiple visits and more in-depth communication [3]. When translated clinical forms do exist, they are usually available in a limited number of languages [7, 8]. For example, research suggests only 27% of medical clinics offer translated documents in Spanish [10], and only 7% of dental clinics provide printed translations in a language other than Spanish [8]. To overcome these language barriers, most private dental clinics use ad hoc interpreters because they are free and readily available [8, 9]. However, some healthcare institutions have relied on in-house translation services provided by bilingual employees and staff translators [12, 13]. In the United States, anyone who is bilingual can provide and certify a translation [14]. At times, interpretation services are provided by bilingual medical students [9, 15, 16]. Although LEP patients prefer to be treated by clinicians who speak the same language they do [17], diversity in health professionals does not match the general population [18, 19]. For example, 75% of dentists are White, 6% Hispanic, 3% Asian, and 2% other races [18], but the general US population is 61% White, 20% Hispanic, 6% Asian, and 8% other races [1].

Even when translations are provided, non-English speakers may not understand the information because they cannot read well in their own language [20]. In a study evaluating the confidence of LEP patients when reading medical forms, 42% needed help with reading [21]. Since low literacy levels are common among non-English speakers, one study investigated literacy on 3 different scales and found that ethnically diverse participants scored lower than White participants [22]. Overall, Hispanic participants scored the lowest, but other non-English and non-Spanish speakers were considered nonliterate in English [22]. In another study, patients were provided with a Spanish translation of drug information, but only 29% understood the document and others could not participate because they did not know how to read [23]. Such difficulties with reading are also compounded by inadequate health literacy. In a study by Hamilton et al. [24], patient knowledge of medical and dental terms commonly used in oral surgery and oral medicine varied from poor to adequate, and the most significant predictor of oral health literacy was knowledge of English. In another study, poor reading ability in the participant’s native language affected their understanding of translated forms [20].

Despite the importance of language services in healthcare for communication, quality of care, and patient satisfaction [17], studies investigating patient perceptions of the readability, understanding, and effect of written materials on patient-provider communication and healthcare experiences are scarce. However, a survey study of LEP patient perceptions reported Spanish speakers were less likely to be satisfied with their healthcare services compared to native English speakers because of inadequate language assistance and a lack of translated written instructions in the patient’s preferred language [25]. Therefore, the purpose of the current study was to investigate the perceptions of LEP dental patients regarding the readability, understanding, and helpfulness of translated clinical forms produced by dental student doctor translators. We hypothesized that the forms would be perceived as helpful and beneficial and that we would find positive correlations between study outcomes.

## Methods

### Participants

The current study used a survey design and convenience sampling to recruit LEP patients from a dental school clinic. The study was conducted from May 2022 to August 2022, and potential participants seeking dental treatment at the clinic had to be aged 18 years or older. Four types of translated clinical forms (consent for treatment, dental health history, no-show policy, and urgent care agreement) were available at the clinic in Arabic, Dari, Pashto, Russian, Spanish, Ukrainian, Urdu, or Vietnamese. Only LEP patients who received forms in one of those languages were invited to participate in the study. Patients who met the inclusion criteria were informed about the study and told that they could withdraw at any time without penalty. Before participation, patients completed a translated and approved informed consent form. The participants were not compensated for their participation. The current study was granted exempt status by the A.T. Still University-Kirksville Institutional Review Board (no. RM20220505).

### Translated clinical forms

As part of a grant-funded pilot project, the bilingual clinical forms used in the current study were translated by calibrated bilingual dental student doctors under the supervision of a bilingual faculty member experienced in translation. Specifically, the translated forms—consent for treatment, a dental and health history questionnaire, the clinic’s no-show policy, and the clinic’s urgent care agreement—provided patients with information about their clinical care or requested health information to provide care. Accuracy of translations were verified by a second bilingual speaker, who was either a dental faculty member or other healthcare professional. To evaluate readability and understanding of the translated forms, we wanted to determine the perceived readability of the translated forms, how the self-reported literacy level of participants affected the readability of the forms, the effect of the forms on participants’ comfort level with receiving dental services at the clinic, how understanding of medical and dental terms affected overall understanding of the translated forms, and the effects of translated forms on the perceived quality of communication between LEP patients and the clinic’s dental care providers. For the current study, readability (legibility) was defined solely as the ability of the participant to read the translated form and the ease of perception of the text. To determine an appropriate sample size for the study, a G*Power calculator was used. For a power of 95% with an α-level of 0.05, a minimum sample size of 47 was calculated.

### Study survey and distribution

A 2-part, 9-question bilingual paper survey was used in the current study to assess the translated clinical forms. Eight items used 5-point Likert-type responses, and 1 item was an open-ended question that requested feedback to improve the translated content of the clinical forms. The Likert-type questions were intended to assess the type of form received; self-reported literacy; design, readability, and helpfulness of the form; overall understanding of the form; understanding of medical and dental terms; helpfulness for patient-provider communication; and comfort level with dental care after receiving the form. The bilingual format of the survey allowed the study researchers to decipher the Likert-type responses regardless of the participant’s native language. Responses to the open-ended question were translated to English by members of the translation team and added to the data.

The survey used in the current study was a slightly modified version of a survey that was designed by one of the study researchers. It was previously validated using factor and correlational analyses (data unpublished). All factor loading estimates were greater than or equal to 0.60, which indicated the variables were good predictors of the factors they were loading to, and the Cronbach α was 0.80, which indicated a good level of interrelatedness. The paper survey was distributed to eligible participants and took about 10 min to complete. Participants could complete the survey more than once for each type of translated form received at the dental appointment.

Before data collection, a calibration session was conducted with dental students, faculty, and staff involved with the study. Privacy and confidentiality policy information was reviewed, and data collection described. Study protocols, including how to recruit participants and distribute the survey, were also discussed. New patients and individuals accessing urgent care services received surveys with translated forms on their arrival during registration. Patients returning to the clinic for care were given translated forms with surveys, if updates were needed. Completed surveys were collected at a central location in the clinic from a locked drop box that was accessible by only one of the study researchers. Patient identification numbers were obtained from completed surveys and used by the researcher to access the clinic’s electronic health record data for the following demographic information about participants: age, gender, preferred language, dentition (teeth or no teeth), intake dental department (comprehensive, oral surgery, periodontal surgery, urgent care), and patient status (existing patient or new patient). Information was also collected about which type of clinical form (consent for treatment, dental health history, no-show policy, and urgent care agreement) was provided to the participant. All study data were entered into a password-protected Google sheet on a password-protected computer.

### Statistical analysis

Demographic characteristics and Likert-type survey responses were summarized using frequency and percentage. Age was summarized using mean and standard deviation. Although translated forms were available in 8 languages, participants asked for forms in only 5 languages. Further, the majority of participants asked for translated forms in only 2 languages (Arabic or Spanish), so the survey responses for the other 3 languages were grouped into an other category for analysis. Spearman correlation analysis was used to determine relationships between survey response outcomes. SPSS statistical software was used for analyses, and a *P* < .05 was considered significant. Responses to the open-ended survey question were translated into English, recorded on data sheet, and entered into QDA Miner Lite qualitative data analysis software. For analysis of these written responses, we decided to use the QDA Miner Lite software even though this aspect of our study design did not involve typical interview qualitative data. Responses were categorized and coded to identify common themes, and the frequency of coded responses was summarized.

## Results

Ninety-seven LEP patients from our dental school clinic participated in the current study. Although our translated clinical forms and the study survey were available in 8 languages, only patients speaking Arabic, Pashto, Spanish, Urdu, and Vietnamese participated. The mean (SD) age of participants was 42 (SD = 10.9) years. The majority of participants were female (61.9%, 60/97), Spanish speakers (78.4%, 70/97), and existing patients (64.9%, 63/97) (Table 1). Most participants visited the comprehensive care department (81.4%, 79/97) for treatment.

**Table 1:** Demographic Characteristics of Study Participants (N = 97)

Demographic Characteristic	No. (%)
Gender	
Female	60 (61.9)
Male	37 (38.1)
Language	
Arabic	15 (15.5)
Pashto	1 (1.0)
Spanish	76 (78.4)
Urdu	1 (1.0)
Vietnamese	4 (4.1)
Dentition	
Dentulous (teeth)	93 (95.9)
Edentulous (no teeth)	4 (4.1)
Dental intake department	
Comprehensive	79 (81.4)
Oral surgery	1 (1.0)
Periodontal surgery	1 (1.0)
Urgent care	16 (16.5)
Patient status	
Existing patient	63 (64.9)
New patient	34 (35.1)

The study survey was completed 140 times by study participants. The dental health history (82.1%, 115/140) was the most frequently received clinical form (Table 2). Although most participants self-reported their reading literacy as very well (57.9%, 81/140) or well (33.6%, 47/140), some (5.0%, 7/140) reported no reading ability. Almost all participants rated the design of the form as excellent (50.4%, 70/140) or good (46.0%, 64/140), the readability as very easy (60.7%, 85/140) or easy (32.9%, 46/140), and the helpfulness as extremely (52.9%, 74/140) or very helpful (38.6%, 54/140). The majority of participants had an overall understanding of the form (67.6%, 94/140) and an understanding of the medical and dental terms used in the form (59.0%, 82/140). Over half (52.9%, 74/140) felt the translated form was extremely helpful for patient-provider communication. Most participants (80.0%, 112/140) indicated their comfort level with dental care was much better after receiving the translated form.

**Table 2:** Survey Responses of Study Participants by Language

Survey Item and Response Option	No. (%)^a^			
	Overall (N = 140)	Spanish (n = 117)	Arabic (n = 17)	Other^b^ (n = 6)
Type of form				
Consent for treatment	10 (7.1)	9 (7.7)	1 (5.9)	0 (0)
Dental health history	115 (82.1)	95 (81.2)	15 (88.2)	5 (83.3)
No-show policy	12 (8.6)	10 (8.5)	1 (5.9)	1 (16.7)
Urgent care agreement	3 (2.1)	3 (2.6)	0 (0)	0 (0)
Self-reported literacy				
Very well	81 (57.9)	67 (57.3)	13 (76.5)	1 (16.7)
Well	47 (33.6)	39 (33.3)	4 (23.5)	4 (66.7)
Somewhat	1 (0.7)	1 (0.9)	0 (0)	0 (0)
With help	4 (2.9)	3 (2.6)	0 (0)	1 (16.7)
Not at all	7 (5.0)	7 (6.0)	0 (0)	0 (0)
Design of form				
Excellent	70 (50.4)	57 (49.1)	12 (70.6)	1 (16.7)
Good	64 (46.0)	56 (48.3)	4 (23.5)	4 (66.7)
Average	5 (3.6)	3 (2.6)	1 (5.9)	1 (16.7)
Poor	0 (0)	0 (0)	0 (0)	0 (0)
Very Poor	0 (0)	0 (0)	0 (0)	0 (0)
Readability of form				
Very easy	85 (60.7)	71 (60.7)	13 (76.5)	1 (16.7)
Easy	46 (32.9)	39 (33.3)	3 (17.6)	4 (66.7)
Neutral	4 (2.9)	3 (2.6)	1 (5.9)	0 (0)
Difficult	4 (2.9)	3 (2.6)	0 (0)	1 (16.7)
Very difficult	1 (0.7)	1 (0.9)	0 (0)	0 (0)
Helpfulness of form				
Extremely	74 (52.2)	62 (53.0)	12 (70.6)	0 (0)
Very	54 (38.6)	45 (38.5)	5 (29.4)	4 (66.7)
Moderate	6 (4.3)	5 (4.3)	0 (0)	1 (16.7)
Somewhat	6 (4.3)	5 (4.3)	0 (0)	1 (16.7)
Not at all	0 (0)	0 (0)	0 (0)	0 (0)
Overall understanding of form				
All	94 (67.6)	80 (69.0)	14 (82.4)	0 (0)
Most	28 (20.1)	23 (19.8)	3 (17.6)	2 (33.3)
Some	12 (8.6)	9 (7.8)	0 (0)	3 (50.0)
Few	5 (3.6)	4 (3.4)	0 (0)	1 (16.7)
None	0 (0)	0 (0)	0 (0)	0 (0)
Understanding of medical and dental terms				
All	82 (59.0)	69 (59.5)	13 (76.5)	0 (0)
Most	37 (26.6)	33 (28.4)	2 (11.8)	2 (33.3)
Some	9 (6.5)	7 (6.0)	1 (5.9)	1 (16.7)
Few	11 (7.9)	7 (6.0)	1 (5.9)	3 (50.0)
None	0 (0)	0 (0)	0 (0)	0 (0)
Helpfulness for patient-provider communication				
Extremely	74 (52.9)	62 (53.0)	12 (70.6)	0 (0)
Very	54 (38.6)	45 (38.5)	5 (29.4)	4 (66.7)
Moderate	6 (4.3)	5 (4.3)	0 (0)	1 (16.7)
Somewhat	6 (4.3)	5 (4.3)	0 (0)	1 (16.7)
Not at all	0 (0)	0 (0)	0 (0)	0 (0)
Comfort level with dental care after receiving form				
Much better	112 (80.0)	94 (80.3)	15 (88.2)	3 (50.0)
Somewhat better	20 (14.3)	18 (15.4)	1 (5.9)	1 (16.7)
Stayed the same	8 (5.7)	5 (4.3)	1 (5.9)	2 (33.3)
Somewhat worse	0 (0)	0 (0)	0 (0)	0 (0)
Much worse	0 (0)	0 (0)	0 (0)	0 (0)

^a^Although only 97 patients participated in the study, they could complete the survey more than once during the study (N = 140 overall survey responses)

^b^Because the majority of surveys were completed for Arabic and Spanish translated forms, surveys completed for Pashto, Urdu, and Vietnamese translated forms were grouped into an other category for analysis

Self-reported literacy and readability of the translated form were positively correlated (Spearman *r* = .57, *P* < .001), but the type of form and readability were not (Spearman *r* = .9, *P* = .36). There was also no correlation between type of form and comfort level with dental care after receiving the form (Spearman *r* = .13, *P* = .18). Overall understanding of the translated form and understanding of medical and dental terms in the form (Spearman = 0.58, *P* < .001) and type of form and helpfulness for patient-provider communication (Spearman *r* = .26, *P* = .005) were also positively correlated. All remaining comparisons were not correlated (all *P* > .05) (Table 3).

**Table 3:** Spearman Correlation Analysis of Likert-Like Survey Questions With Associated 95% Confidence Intervals

Question	Question								
	1	2	3	4	5	6	7	8	9
1. How well do you read in your primary language?	-								
2. How would you rate the design of the form	0.31*** 0.15 to 0.48	-							
3. How easy was it to read the form in your preferred language?	0.57*** 0.32 to 0.61	0.55*** 0.39 to 0.65	-						
4. How helpful was it to have the form translated?	0.48*** 0.19 to 0.51	0.46*** 0.24 to 0.55	0.69*** 0.50 to 0.72	-					
5. How many words written in your preferred language did you understand?	0.47*** 0.52 to 0.74	0.50*** 0.28 to 0.58	0.61*** 0.32 to 0.61	0.52*** 0.27 to 0.57	-				
6. How many dental or medical terms written in your preferred language did you understand?	0.25** 0.16 to 0.49	0.61*** 0.45 to 0.69	0.49*** 0.37 to 0.64	0.47*** 0.24 to 0.55	0.58*** 0.34 to 0.62	-			
7. How helpful was the form for communication and level of understanding between you and the dental provider?	0.26** 0.20 to 0.52	0.65*** 0.40 to 0.66	0.55*** 0.27 to 0.57	0.57*** 0.28 to 0.58	0.52*** 0.39 to 0.66	0.68*** 0.55 to 0.76	-		
8. After reading the translated form, how has your comfort level with receiving dental care changed?	0.14 −0.09 to 0.27	0.33*** 0.17 to 0.49	0.40*** 0.14 to 0.47	0.50*** 0.41 to 0.67	0.50*** 0.31 to 0.60	0.30** 0.06 to 0.40	0.34*** 0.13 to 0.46	-	
9. Form	0.30** 0.19 to 0.51	0.33*** 0.12 to 0.45	0.09 −0.16 to 0.20	0.17 −0.03 to 0.32	0.22* 0.05 to 0.40	0.18 −0.03 to 0.33	0.26** 0.11 to 0.45	0.13 −0.12 to 0.24	-

***P < .001, **P < .01, *P < .05

Correlation is significant at *P* < .05

Of the 97 participants who completed 140 surveys, only 20 (21%) responded to the open-ended question asking for feedback to improve the translated clinical forms (Table 4). Of those, most (95%, 19) were native Spanish speakers; 1 (5%) spoke Arabic. Written responses indicated these participants were satisfied with the quality of the translated forms and believed the translations were good (19 responses); some indicated that no improvements or corrections were needed (8 responses) and that they considered the translations as great customer service (1 response).

**Table 4:** Analysis of Participant Written Responses to the Open-Ended Survey Question (N = 20)^a^

Category and Code	No. Responses by Code^b^
Quality of form	
Good (Spanish speaker)	18
Good (Arabic speaker)	1
Corrections needed	
No corrections needed (Spanish speaker)	1
Customer service contribution	
Great customer service (Spanish speaker)	1
Improvements needed	
No improvements needed (Spanish speaker)	6
No improvements needed (Arabic speaker)	1
Understanding of text	
Did not understand acronym	1

^a^Nineteen participants spoke Spanish, and 1 spoke Arabic

^b^The number of responses by code may not represent all 20 participants because some responses may not have been coded under the specific categories reported in the table

## Discussion

The current study investigated the perceptions of LEP dental patients regarding the readability, understanding, and helpfulness of translated clinical forms produced by dental student doctor translators. To our knowledge, the current study is the first to investigate patient perceptions of the helpfulness and readability of bilingual clinical forms in a dental school clinical setting. Our results suggested the translated forms were perceived as effective and beneficial for patient satisfaction, comfort level with dental care, and helpfulness for patient-provider communication. These findings were supported by other studies investigating the effects of language assistance on patient care [26]. Further, the majority of LEP patients in the current study were satisfied with the translations produced by our dental students, which agreed with previous research suggesting that multilingual healthcare professionals and medical students are qualified to provide language assistance [14, 16].

As indicated in previous studies [20, 22], the poor understanding of our translated forms by some study participants may have been caused by low literacy and lack of health literacy in any language. Although most participants reported readability of the forms was good, those who rated the readability as difficult or very difficult may have needed help with reading. Our analysis of survey outcomes found positive and statistically significant correlations between self-reported literacy and readability and between type of form and helpfulness for patient-provider communication. These findings further support the importance of reading literacy for overall understanding of translated text. Given these results, healthcare providers should be mindful LEP patient literacy levels and try to identify those with poor literacy. Such patients may need additional verbal language assistance to better understand translated clinical forms.

The current study also found a significant correlation between overall understanding of the translated forms and understanding of medical and dental terms in the forms. Similarly, a study by Oliva [20] reported that low health literacy negatively affects overall understanding of clinical materials. Other studies have reported low health and oral health literacy in adult populations [22, 24], which is likely more common among linguistically diverse individuals. Although some of our participants did not understand the medical and dental terms in the forms, the significant correlation related to helpfulness of the forms for patient-provider communication likely had a positive effect on the comfort and stress levels LEP patients, as suggested by similar studies [17].

Given the growing diversity of the US population [1] and the negative impact of language barriers on the health of LEP patients [3, 4], results of the current study may support future development of language assistance programs for these patients. Our innovative approach of using bilingual dental students as translators of clinical forms may be a plausible solution to increase the number of translated clinical forms, reduce the costs associated with translation services, and bridge the gap in written language assistance that currently exists in dentistry [3, 4, 7–9]. The positive survey responses and feedback from our study participants seemed to support the notion that a diverse healthcare workforce is qualified to provide language assistance [12]. Therefore, recruiting bilingual employees and using nonverbal communication methods may be one of the ways to address communication barriers in healthcare [3, 8].

### Limitations

The current study had several limitations. Our use of convenience sampling and the limited number of translated clinical forms may have introduced selection bias, thus affecting the generalizability of findings. Nonresponse bias and self-reported bias may have also affected the external validity of results. When performing survey studies, question order bias may be problematic. To address this potential limitation, we reversed the order of Likert-type response options in the last survey item of that type. Another limitation was related to distribution of the survey to patients accessing the clinic for care. Initially, we anticipated that the majority of participants would arrive through the clinic’s urgent care department. During the study, that department experienced staff shortages and temporarily closed. As a result, many of the participants were returning patients who did not always receive translated forms that are typically given to new patients, which likely reduced the number of completed surveys. Once this problem was identified, the project director reviewed each day’s clinic schedule to identify returning patients who needed updated forms, ensuring that surveys were readily available and that the treating clinicians distributed the translated forms and survey to potential study participants.

### Recommendations

Given results of the current study, we recommend additional research on this topic in a variety of dental and healthcare settings where bilingual clinicians are employed. Studies should also investigation the effects of a greater variety of languages for translated clinical forms to increase external validity and generalizability of results. In addition, future studies should investigate the overall health literacy and the oral health literacy of LEP patients. The development and application of interventional programs that increase patient literacy in these areas may lead to better patient outcomes.

## Conclusion

In the current study, the perceptions LEP patients regarding clinical forms translated by bilingual dental students were overwhelmingly positive. While most innovative language assistance programs in healthcare are concerned with interpretation, our results illustrated a cost-effective way of providing translations for LEP patients. Many dental schools struggle to identify funding for language assistance, so innovative pilot projects using the bilingual talents of students and faculty, as in the current study, should be further explored. A model similar to that used in our study could be easily introduced in other healthcare education programs that have a linguistically diverse student population, funding for language assistance, and a mechanism for training and supervision in translation.

## Data Availability

The datasets used and/or analyzed during the current study are available from the corresponding author on reasonable request.

## Abbreviations

LEP: Limited English proficiency
